# Supplementary material for: Epitranscriptional m6A modification of rRNA negatively impacts translation and host colonization in Staphylococcus aureus
Source: PLoS Pathog. 2024 Jan 22;20(1):e1011968. doi: 10.1371/journal.ppat.1011968 (PMC10833563; doi:10.1371/journal.ppat.1011968)
Supplement: S1 Table — Minimum inhibitory concentration (MIC, in μg/mL) were determined by E-test on Muller Hinton Agar plates in duplicates per strain per antibiotic type. (PDF) [file ppat.1011968.s001.pdf]

S1 Table. Removal of *ermBL* coding sequence from the *ermBL-ermB* operon renders low levels of macrolide resistance. Minimum inhibitory concentration (MIC, in µg/mL) were determined by E-test on Muller Hinton Agar plates in duplicates per strain per antibiotic type.

**Cellular targets**	**Abx**[1]	**Parental JE2**	**JE2 *att*:pJC1111: P_{erm}-*ermB*-$CdCl_2^R$**		**JE2 *att*:pJC1306 : P_{erm}-*ermB*-Tet^R**	
			ermB^{WT}	***ermB***^{Y103A}	***ermB***^{WT}	***ermB***^{Y103A}
**50S ribosomal subunit**	**ERY**	0.19	0.5	0.094	0.5	0.064
	**CLN**	0.38	0.5	0.064	0.125	0.047
	**SOL**	0.064	0.064	0.064	0.064	0.064

[1] Antibiotics (Abx); Erythromycin (ERY); Clindamycin (CLN); Solithromycin (SOL).
