# Supplementary material for: Epitranscriptional m6A modification of rRNA negatively impacts translation and host colonization in Staphylococcus aureus
Source: PLoS Pathog. 2024 Jan 22;20(1):e1011968. doi: 10.1371/journal.ppat.1011968 (PMC10833563; doi:10.1371/journal.ppat.1011968)
Supplement: S2 Table — (PDF) [file ppat.1011968.s002.pdf]

**S2 Table. Summary of Ribo-seq and mRNA-seq libraries reported in this study.**

| replicate | Sample name[a] | 23S rRNA status[b] | Sequencing type[c] | Mapped reads[d] | Replicates correlation | R square[e] |
|---|---|---|---|---|---|---|
| Rep 1 | YP2 | A2058 | RPF | 10,171,206 | | |
| Rep 1 | YP4 | A2058 | Total mRNA | 3,338,508 | | |
| Rep 2 | YP6 | A2058 | RPF | 4,238,333 | vs. YP2 | 0.9526 |
| Rep 2 | YP8 | A2058 | Total mRNA | 4,899,691 | vs. YP4 | 0.9426 |
| Rep 3 | YP10 | A2058 | RPF | 11,204,278 | vs. YP2 | 0.9424 |
| Rep 3 | YP12 | A2058 | Total mRNA | 5,299,356 | vs. YP4 | 0.9402 |
| Rep 1 | YP13 | $m^6_2$A2058 | RPF | 19,648,917 | | |
| Rep 2 | YP15 | $m^6_2$A2058 | RPF | 10,660,958 | vs. YP13 | 0.9897 |
| Rep 1 | YP16 | $m^6_2$A2058 | Total mRNA | 9,640,961 | | |
| Rep 2 | YP18 | $m^6_2$A2058 | Total mRNA | 4,287,057 | Vs. YP18 | 0.9485 |

[a] corresponding to sample labels reported in GEO accession GSE168265
[b] 23S rRNA modification status, all strains from parental *S. aureus* USA300 JE2.
[c] RPF, ribosome-protected footprints
[d] unique reads that mapped to the *S. aureus* USA300 genome (GenBank CP000255.1) using Bowtie2, excluding rRNA and tRNA. Two mismatches were allowed.
[e] squared correlation coefficient of replicate 1 and 2 from the same sample type.
