# Supplementary material for: Epitranscriptional m6A modification of rRNA negatively impacts translation and host colonization in Staphylococcus aureus
Source: PLoS Pathog. 2024 Jan 22;20(1):e1011968. doi: 10.1371/journal.ppat.1011968 (PMC10833563; doi:10.1371/journal.ppat.1011968)
Supplement: S3 Table — (PDF) [file ppat.1011968.s003.pdf]

S3 Table. Strains and plasmids.

Name	Genotypes and relevant features	Source
S. aureus		
JE2	Parental strain, plasmid cured LAC USA300 strain	BEI resources [1]
RN4220	*sau1⁻*, *hsdR⁻*, *mec⁻*, *rsbU⁻*, *agr⁻*, plasmid passage host	ATCC NR-45946
RN4220Δ*spa*	Δ*spa::*Erm	[2]
RN9011	RN4220/pRN7023, SaPI1 integrase, *cat194*	[3]
NCTC 8325	Septic patient isolate in 1960, *sasG*$^{+}$	NARSA repository
KES18	JE2 *att::ermB*^{WT}, $CdCl_2$ ^{R}	This study
KES19	JE2 *att::ermB*^{Y103A}, $CdCl_2$ ^{R}	This study
KES25	JE2 *att::ermB*^{WT}, Tet^{R}	This study
KES26	JE2 *att::ermB*^{Y103A}, Tet^{R}	This study
KES29	JE2 *att:: ermBL*^{WT}-*ermB*^{WT}, $CdCl_2$ ^{R}	This study
KES30d	JE2 *att:: ermBL*^{R7stop}-*ermB*^{WT}, $CdCl_2$ ^{R}	This study
KES34	JE2 *att:: ermBL*^{WT}-*ermB*^{Y103A}, $CdCl_2$^{R}	This study
MNY196	JE2 *att:: ermBL*^{R7stop}-*ermB*$^{1I75T/N100S}$, $CdCl_2$^{R}	This study
KES61	JE2 *att:: ermBL*^{WT}-*ermB*^{WT}, Tet^{R}	This study
KES59a	JE2 *att:: ermBL*^{R7stop}-*ermB*^{WT}, Tet^{R}	This study
NE1241	JE2 Δ*nuc2::*Erm	BEI resources
NR-46641	JE2 Δ*sdrE*::Erm	BEI resources
NE286	JE2 Δ*spa::*Erm	BEI resources
NE1109	JE2 Δ*sigB*::Erm	BEI resources
NE825	JE2 Δ*sasG(5')*::Erm	BEI resources
NE361	JE2 Δ*ywlG*::Erm	BEI resources
E. coli		
BL21(DE3)	F⁻ *ompT gal dcm lon hsdS*$_{B}$(*r*$_{B}^{-}$*m*$_{B}^{-}$) λ(DE3 [*lacI lacUV5-T7p07 ind1 sam7 nin5*]) [*malB*$^{+}$]$_{K-12}$(λ^{S})	Lucigen
DC10B	DH10B Δ*dcm*	[4]
**Plasmids**		
pLI50	*E. coli-S. aureus* shuttle vector, promoterless Amp^{R}, Cm^{R}	[5]
pZS-P$_{tet}$-mcherry-yfp (WT)	*mcherry-yfp* (WT) , Amp^{R}	[6]
pZS-P$_{tet}$-mcherry-yfp (TGA)	*mcherry-yfp* (TGA) , Amp^{R}	[6]
pZS-P$_{tet}$-mcherry-yfp (TAG)	*mcherry-yfp* (UAG) , Amp^{R}	[6]
pZS-P$_{tet}$-mcherry-yfp (TAA)	*mcherry-yfp* (UAA) , Amp^{R}	[6]
pLI50-m-y [WT]	*mcherry-yfp* containing P$_{hpf}$ promoter on pLI50, Amp^{R}, Cm^{R}	This work
pLI50-m-y [TGA]	*mcherry-yfp*(UGA) containing P$_{hpf}$ promoter on pLI50, Amp^{R}, Cm^{R}	This work
pLI50-m-y TAG]	*mcherry-yfp*(UAG) containing P$_{hpf}$ promoter on pLI50, Amp^{R}, Cm^{R}	This work
pLI50-m-y [TAA]	*mcherry-yfp*(UAA) containing P$_{hpf}$ promoter on pLI50, Amp^{R}, Cm^{R}	This work
pLI50-m-y [-1 fs]	*mcherry-yfp*(-1 fs) containing P$_{hpf}$ promoter on pLI50, Amp^{R}, Cm^{R}	This work

pLI50-m-y [+1 fs]	*mcherry-yfp*(+1 fs) containing P_{hpf} promoter on pLI50, Amp^R, Cm^R	This work
pJC1111	SaPI1 *attS* suicide vector, Amp^R, $CdCl_2{}^R$	[3]
pJC1306	SaPI1 *attS* suicide vector, Amp^R, Tet^R	[3]
pGEMT-Easy	T-A cloning vector, Amp^R	Promega
pTA_P_{erm} *ermBL*^{WT}-*ermB*^{WT}	pGEMT-Easy carrying the full *ermBL-ermB*	This work
pTA_P_{erm} *ermBL*^{WT}-*ermB*^{Y103A}	Catalytically inactive $ErmB^{Y103A,}$ Amp^R	This work
pTA_P_{erm} *ermBL*^{R7stop}-*ermB*^{WT}	Constitutively expressed $ErmB^{WT}$, Amp^R	This work
pTA_P_{erm} *ermBL*^{R7stop}-*ermB*$^{I75T/N100S}$	Constitutively expressed $ErmB^{I75T/N100S}$ with an increase hypermethylation activity, Amp^R	This work
pLI50P_{erm}-*ermB*^{WT}	*ermB*^{WT} without *ermBL* cds, Amp^R (*E. coli*), Cm^R (*S. aureus*)	This work
pLI50P_{erm}-*ermB*^{Y103A}	*ermB*^{Y103A} without *ermBL* cds, Amp^R (*E. coli*), Cm^R (*S. aureus*)	This work
pJC1111:: *ermBL*^{WT}-*ermB*^{WT}	*ermBL*^{WT}-*ermB*^{WT}, Amp^R (*E. coli*), $CdCl_2$ (*S. aureus*)	This work
pJC1111:: *ermBL*^{WT}-*ermB*^{Y103A}	*ermBL*^{WT}-*ermB*^{Y103A}, Amp^R (*E. coli*), $CdCl_2$ (*S. aureus*)	This work
pJC1111:: *ermBL*^{R7stop}-*ermB*^{WT}	*ermBL*^{R7stop}-*ermB*^{WT}, Amp^R (*E. coli*), $CdCl_2$ (*S. aureus*)	This work
pJC1111:: *ermBL*^{R7stop}-*ermB*$^{I75T/N100S}$	*ermBL*^{R7stop}-*ermB*$^{I75T/N100S}$, Amp^R (*E. coli*), $CdCl_2$ (*S. aureus*)	This work
pJC1111:: *ermB*^{WT}	*ermB*^{WT} without *ermBL* cds, Amp^R (*E. coli*), $CdCl_2{}^R$ (*S. aureus*)	This work
pJC1111:: *ermB*^{Y103A}	*ermB*^{Y103A} without *ermBL* cds, Amp^R (*E. coli*), $CdCl_2{}^R$(*S. aureus*)	This work
pJC1306:: *ermBL*^{WT}-*ermB*^{WT}	*ermBL*^{WT}-*ermB*^{WT}, Amp^R (*E. coli*), Tet^R(*S. aureus*)	This work
pJC1306:: *ermBL*^{R7stop}-*ermB*^{WT}	*ermBL*^{R7stop}-*ermB*^{WT}, Amp^R (*E. coli*), Tet^R(*S. aureus*)	This work

1. Fey PD, Endres JL, Yajjala VK, Widhelm TJ, Boissy RJ, Bose JL, et al. A genetic resource for rapid and comprehensive phenotype screening of nonessential *Staphylococcus aureus* genes. mBio. 2013;4(1):e00537-12.
2. Keener AB, Thurlow LT, Kang S, Spidale NA, Clarke SH, Cunnion KM, et al. *Staphylococcus aureus* Protein A Disrupts Immunity Mediated by Long-Lived Plasma Cells. J Immunol. 2017;198(3):1263-73.
3. Chen J, Yoong P, Ram G, Torres VJ, Novick RP. Single-copy vectors for integration at the SaPI1 attachment site for *Staphylococcus aureus*. Plasmid. 2014;76:1-7.
4. Monk IR, Shah IM, Xu M, Tan MW, Foster TJ. Transforming the untransformable: application of direct transformation to manipulate genetically *Staphylococcus aureus* and *Staphylococcus epidermidis*. mBio. 2012;3(2):e00277-11.
5. Lee CY, Buranen SL, Ye ZH. Construction of single-copy integration vectors for *Staphylococcus aureus*. Gene. 1991;103(1):101-5.
6. Fan Y, Evans CR, Barber KW, Banerjee K, Weiss KJ, Margolin W, et al. Heterogeneity of Stop Codon Readthrough in Single Bacterial Cells and Implications for Population Fitness. Mol Cell. 2017;67(5):826-36
