# Supplementary material for: Epitranscriptional m6A modification of rRNA negatively impacts translation and host colonization in Staphylococcus aureus
Source: PLoS Pathog. 2024 Jan 22;20(1):e1011968. doi: 10.1371/journal.ppat.1011968 (PMC10833563; doi:10.1371/journal.ppat.1011968)
Supplement: S4 Table — (PDF) [file ppat.1011968.s004.pdf]

S4 Table. Oligonucleotides used in this study

Primer	Sequence (5'-3')[a]	Application
P823 (SacI)f<br>P824r<br>P825f<br>P826 (HindIII)r	CGAGAGCTCTTGGTCTTGCGTATGGTTA<br>GTTCATGTAATCACTCCTGAAGTGATTTCTTAATTACAAATTTTTAGCAT<br>ATGCTAAAAATTTGTAATTAAGAAATCACTTCAGGAGTGATTACATGAAC<br>CGAAGCTTAGAATTATTTCCTCCCG	Crossover PCR to remove *ermBL* coding region and cloned into the SacI and HindIII sites of pLI50.
P960f<br>P961r	GGATATTCACCGAACACTAGGG<br>TGGAACATCTGTGGTATGGC	RT-qPCR for 3'-*ermB*
P1221f<br>P1222r	CGAAATTGGAACAGGTAAAGGG<br>CTGACGATAAGTTGAATAGATGACTG	RT-qPCR for 5'-*ermB*
P1205f<br>P1206r	CAGGTGACACAGCGGGTATA<br>TGCCGGGTTGTGATGCTATT	RT-qPCR for *polC*
P827f<br>P828r	TGGGAATATTCCTGCCCATTTAAGCACA<br>TGTGCTTAAATGGGCAGGAATATTCCCA	Mutagenesis to create $ermB^{Y103A}$
P885f<br>P886r	GTTGGTATTCCAAATGTAATATGTAGATAAAACATCT<br>AGATGTTTTATCTACATATTACATTTGGAATACCAAC	Mutagenesis to create $ermBL^{R7stop}$
P1269f<br>P1270r	AATTAAAACTGAACACTCGTGTCACTTTAAT<br>ATTAAAGTGACACGAGTGTTCAGTTTTAATT	Mutagenesis to create $ermB^{I75T}$
P1271f<br>P1272r	ATAAAATTGTTGGGAGTATTCCTTACCATTA<br>TAATGGTAAGGAATACTCCCAACAATTTTAT	Mutagenesis to create $ermB^{N100S}$
P1186 (PstI)f<br>P1187 (KpnI)r	ATCTGCAGTTGGTCTTGCGTATGGTTAACCCTAAAG<br>TAGGTACCTAGAATTATTTCCTCCCGTTAAATAATAGA	Cloning of the *ermBL-ermB* with its native promoter
P1189f<br>P1190r	CGGTTCCTGGCCTTTTGCTGGCCTTTTGCT<br>TCAGTATTTATTATGCATTTAGAATAGG	Sequencing primers on pJC1111/pJC1306
P1191f<br>P1192r	GTGCTTCACCAGCACCACATGCTG<br>GTATTAGTTTGAGCTGTCTTGGTTCATTGATTGC	Primers to confirm pJC chromosomal integration.
P651(BamHI)<br>P1444<br>P1445<br>P1446(HindIII)	CGGGATCCATACAACTGGATTAACAATTCATCGTGCAGGGTG<br>CCTCCTCGCCCTTGGAAACCATAGTAATCTCTCCTTAAACCTCTTTAT<br>ATAAAGAGGTTTAAGGAGAGATTACTATGGTTTCCAAGGGCGAGGAGG<br>CAGCTAATTAAGCTTCTATTTATACAGTTCGTCC	Crossover PCR oligos to create a P_{hpf}-driven *mcherry-yfp* reporter on pLI50.
P1539<br>P1540	GATGAGCTGTACAAATACTGCAGACCAGCGCG<br>CGCGCTGGTCTGCAGTATTTGTACAGCTCATC	Mutagenesis to create -1 fs *mcherry-yfp* reporter.
P1541<br>P1542	GATGAGCTGTACAAATATACTGCAGACCAGCGCG<br>CGCGCTGGTCTGCAGTATATTTGTACAGCT CAT C	Mutagenesis to create +1 fs *mcherry-yfp* reporter.
P630<br>P631	GCA CAT TTC CCC GAA AAG TGC CAC CTG ACG T<br>TGC CTT TAT TTT GAA TTT TAA GGG GCA T	pLI50 sequencing primers.
FAM_Sa2058_R1 (P813)	5'[6-FAM]-AGT AAA GCT CCCA CGG GGTC[b]<br>(3-nt downstream of m^6A2058)	Primer extension to normalize m^6_2A2058 signal
FAM_Sa2058_R2 (P1614)	5'[6-FAM]-TCC TGT ACA AGC TGT GCC GAAT[b]	Primer extension to map m^6_2A2058

[a]: restriction enzyme cleavage sites are underlined. [b]: 6-FAM, 6-carboxyfluorescein
