# Supplementary material for: Epitranscriptional m6A modification of rRNA negatively impacts translation and host colonization in Staphylococcus aureus
Source: PLoS Pathog. 2024 Jan 22;20(1):e1011968. doi: 10.1371/journal.ppat.1011968 (PMC10833563; doi:10.1371/journal.ppat.1011968)
Supplement: S1 Fig — (A-C) S. aureus strains (JE2 (ermBL—ermB-), ermBLR7Stop-ermBWT, ermBLR7Stop-ermBY103A, ermBLR7Stop-ermBI75T/N100S) were grown overnight in TSB at 37°C until OD600 = 0.5. Cells were stained with the membrane dye FM4-64 and the DNA dye Hoechst 33342 and visualized on an epifluorescence microscope. The images were acquired in an unbiased manner by using the multiple image alignment function in NIS Element software (Nikon). At least 50 cells distributed on >3 image frames were recorded. Scale bar = 2 μm. (D) Transmission electron microscopy (TEM) analysis detects no significant alteration in cell wall thickness and ultrastructure of S. aureus cells with and without an active ErmB. At least 35 cells distributed on 2 image frames were recorded. Representative single cells are shown. Scale bars represent 100 nm with 49,000× magnification. (PDF) [file ppat.1011968.s006.pdf]

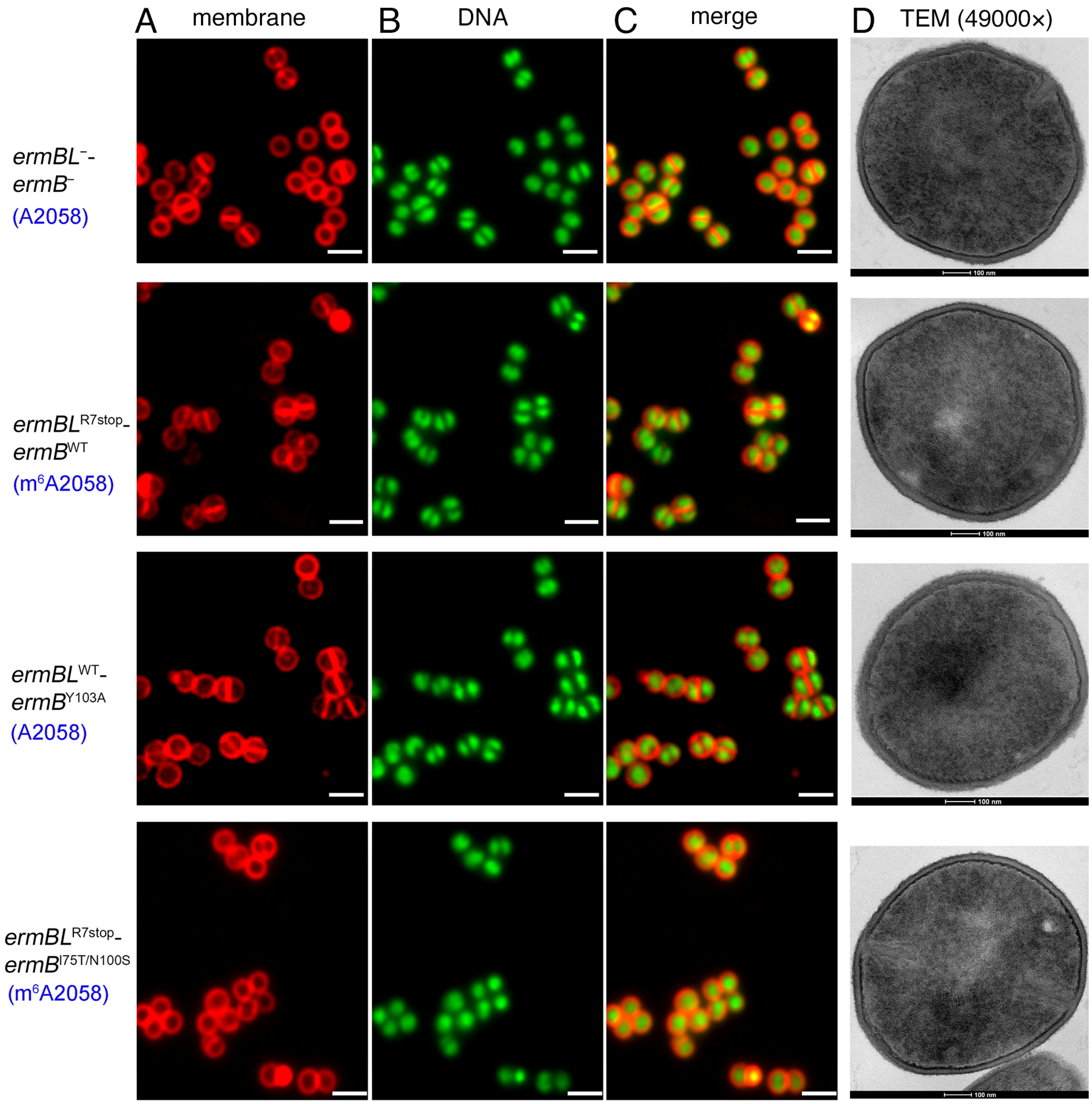

S1 Fig. Cell morphology and cell size were unaltered upon m^{6}A modification of ribosomes. (A-C) *S. aureus* strains (JE2 (*ermBL*$^-$-*ermB*$^-$), *ermBL*^{R7Stop}-*ermB*^{WT}, *ermBL*^{R7Stop}-*ermB*^{Y103A}, *ermBL*^{R7Stop}-*ermB*$^{I75T/N100S}$) were grown overnight in TSB at 37°C until OD_{600} = 0.5. Cells were stained with the membrane dye FM4-64 and the DNA dye Hoechst 33342 and visualized on an epifluorescence microscope. The images were acquired in an unbiased manner by using the multiple image alignment function in NIS Element software (Nikon). At least 50 cells distributed on >3 image frames were recorded. Scale bar= 2 µm. **(D)** Transmission electron microscopy (TEM) analysis detects no significant alteration in cell wall thickness and ultrastructure of *S. aureus* cells with and without an active ErmB. At least 35 cells distributed on 2 image frames were recorded. Representative single cells are shown. Scale bars represent 100 nm with 49,000× magnification.
