# Supplementary material for: Epitranscriptional m6A modification of rRNA negatively impacts translation and host colonization in Staphylococcus aureus
Source: PLoS Pathog. 2024 Jan 22;20(1):e1011968. doi: 10.1371/journal.ppat.1011968 (PMC10833563; doi:10.1371/journal.ppat.1011968)
Supplement: S3 Fig — Ribosome sedimentation profiles (n = 3) of Ribo-seq samples prepared from ermBLWT-ermBY103A and ermBLR7Stop-ermBWT strains were subjected to micrococcal nuclease (MNase) treatment or untreated and analyzed by 10–40% sucrose density gradient ultracentrifugation. The 100S ribosomes do not contain mRNA and are not collapsed into 70S monosomes like the polysomes, showing distinct peaks that are indistinguishable from the translating disomes on a sucrose gradient due to near identical masses. Peak height is indicated by the absorbance at 254 nm (y-axis). Each sedimentation profile contains twenty-five Abs260 units of input crude ribosomes that were isolated from logarithmically grown S. aureus cells (OD600~ 0.9–1.0). (PDF) [file ppat.1011968.s008.pdf]

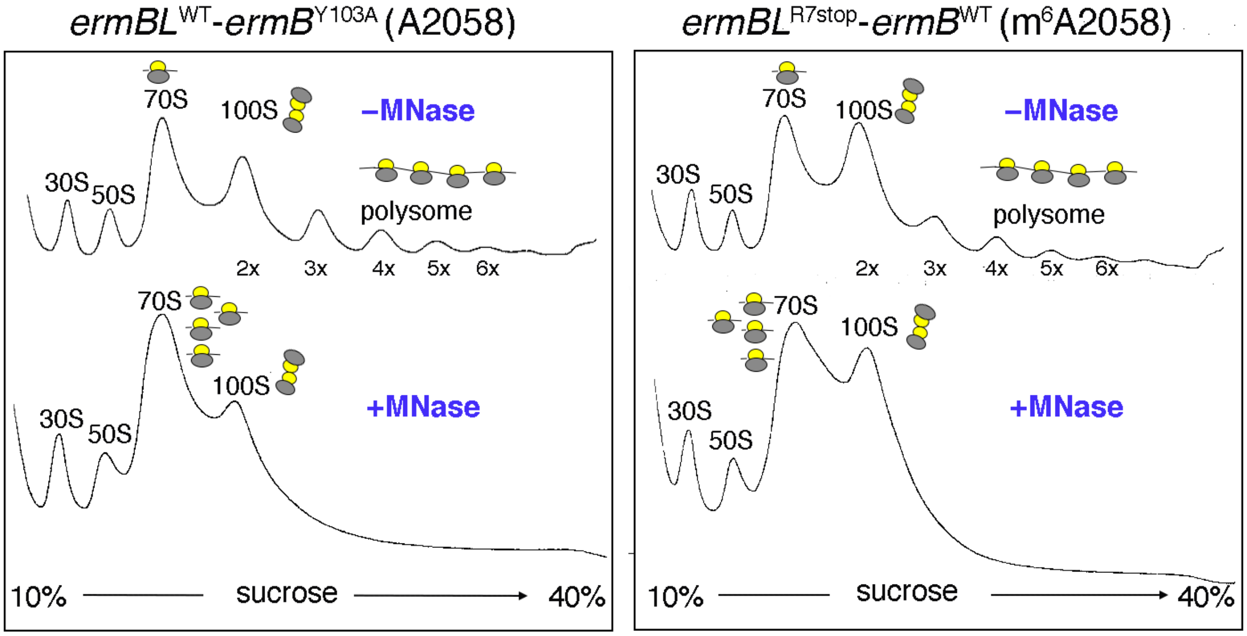

S3 Fig. m^{6}A modification does not affect ribosome assembly but increases the abundance of translationally inactive 100S ribosomes. Ribosome sedimentation profiles (n=3) of Ribo-seq samples prepared from *ermBL*^{WT}-*ermB*^{Y103A} and *ermBL*^{R7Stop}-*ermB*^{WT} strains were subjected to micrococcal nuclease (MNase) treatment or untreated and analyzed by 10-40% sucrose density gradient ultracentrifugation. The 100S ribosomes do not contain mRNA and are not collapsed into 70S monosomes like the polysomes, showing distinct peaks that are indistinguishable from the translating disomes on a sucrose gradient due to near identical masses. Peak height is indicated by the absorbance at 254 nm (y-axis). Each sedimentation profile contains twenty-five Abs$_{260}$ units of input crude ribosomes that were isolated from logarithmically grown *S. aureus* cells (OD$_{600}$~ 0.9-1.0)
