# Supplementary material for: Epitranscriptional m6A modification of rRNA negatively impacts translation and host colonization in Staphylococcus aureus
Source: PLoS Pathog. 2024 Jan 22;20(1):e1011968. doi: 10.1371/journal.ppat.1011968 (PMC10833563; doi:10.1371/journal.ppat.1011968)
Supplement: S4 Fig — (A) Ribosome stalling peptide upstream of ilv-leu amino acid biosynthesis operon. Peptide sequence and stalling motif are highlighted in magenta. (B) +1 programmed frameshift in prfB gene. (C) Translational profile of cidC (formerly poxB). (D) Overall low ribosome occupancy (poor mRNA translation) in tenA. In panels C-D, regions highlighted in green indicate a frameshift site in cidC and a ribosome pausing site in tenA. These events were not observed in this Ribo-seq study likely due to the use of different S. aureus strains. A small RNA s900 is annotated upstream of tenA. (PDF) [file ppat.1011968.s009.pdf]

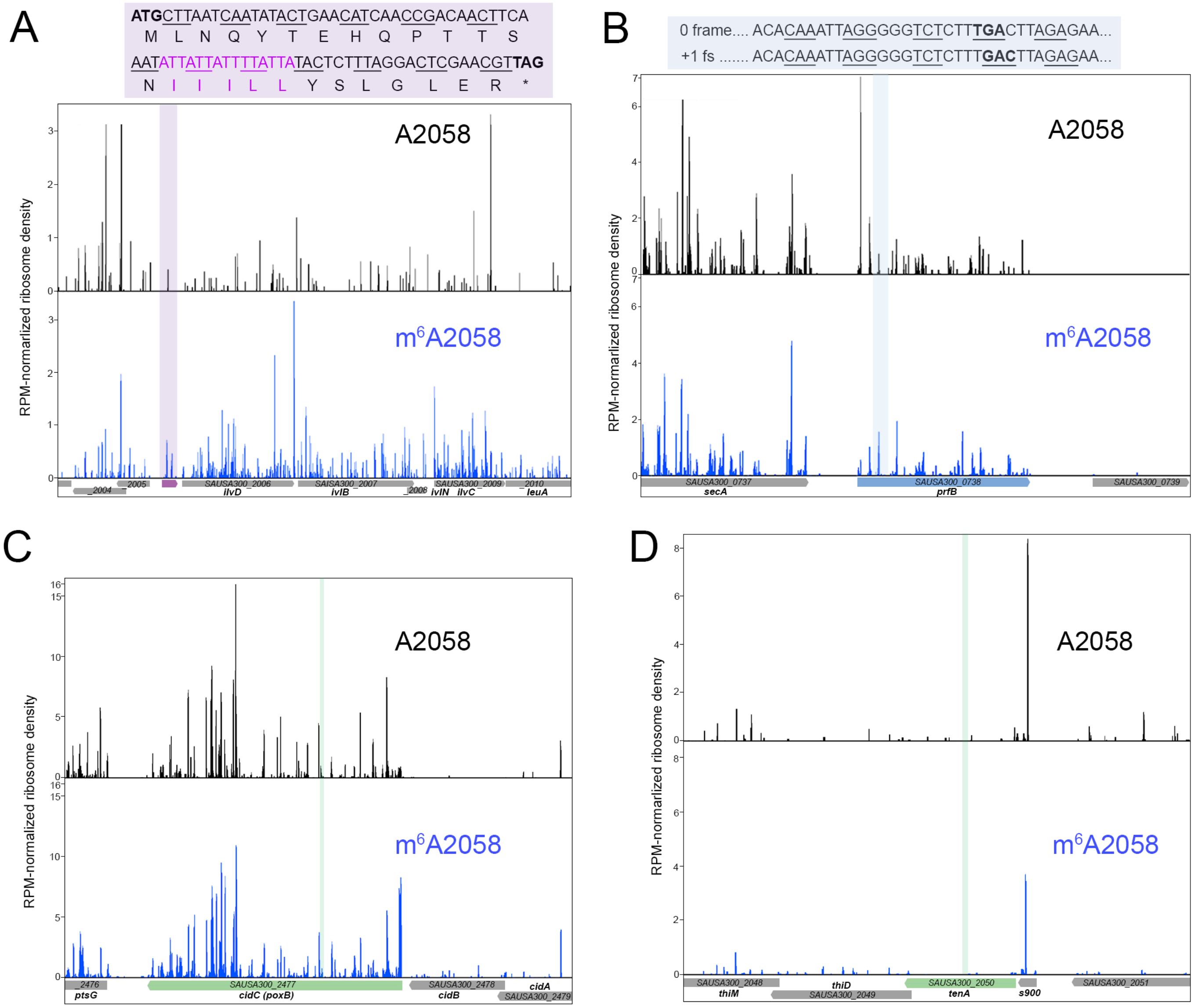

S4 Fig. Ribosome occupancy profile of genes with known frameshift event or ribosome stalling sites and candidate genes identified from previous study [60]. (A) Ribosome stalling peptide upstream of *ilv-leu* amino acid biosynthesis operon. Peptide sequence and stalling motif are highlighted in magenta. **(B)** +1 programmed frameshift in *prfB* gene**. (C)** Translational profile of *cidC* (formerly *poxB*). **(D)** Overall low ribosome occupancy (poor mRNA translation) in *tenA*. In panels C-D, regions highlighted in green indicate a frameshift site in *cidC* and a ribosome pausing site in *tenA.* These events were not observed in this Ribo-seq study likely due to the use of different *S. aureus* strains. A small RNA s900 is annotated upstream of *tenA*.
