# Supplementary material for: Epitranscriptional m6A modification of rRNA negatively impacts translation and host colonization in Staphylococcus aureus
Source: PLoS Pathog. 2024 Jan 22;20(1):e1011968. doi: 10.1371/journal.ppat.1011968 (PMC10833563; doi:10.1371/journal.ppat.1011968)
Supplement: S2 Fig — Primer extension was performed with a fluorescently labeled primer P813 (S4 Table), 250 ng rRNA, and Superscript III reverse transcriptase in the presence of 1 mM dATP, dGTP, 0.25 mM dTTP and 1 mM ddCTP. The cDNA products were resolved on a 10% denaturing polyacrylamide gel. Reverse transcription is impeded by m6A2058. cDNA synthesis terminates at G2056 and serves as a normalization reference. (PDF) [file ppat.1011968.s007.pdf]

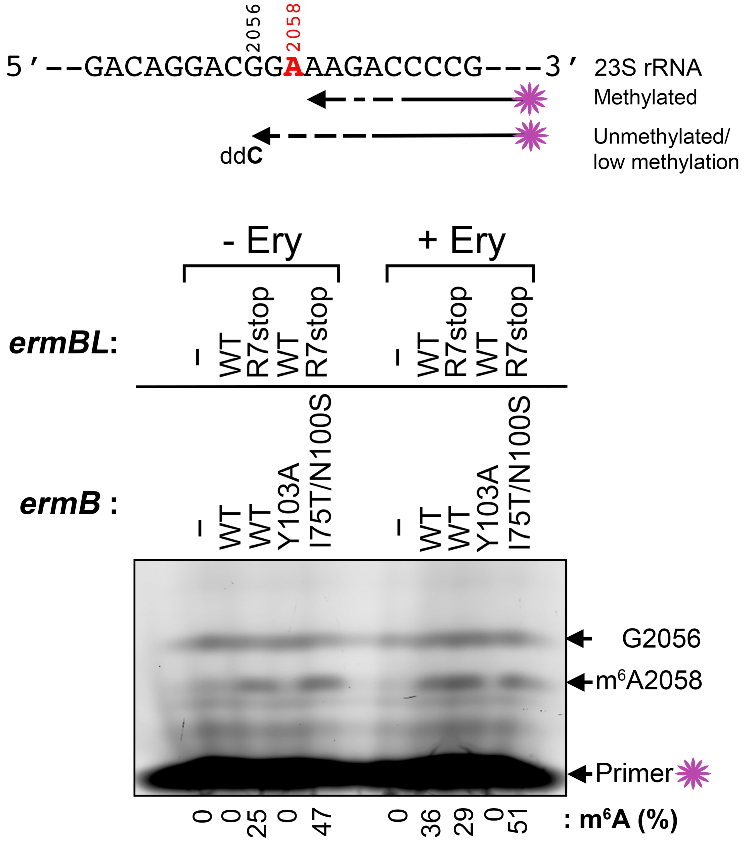

S2 Fig. Estimation of m^{6}A2058 modification by primer extension. Primer extension was performed with a fluorescently labeled primer P813 (S4 Table), 250 ng rRNA, and Superscript III reverse transcriptase in the presence of 1 mM dATP, dGTP, 0.25 mM dTTP and 1 mM ddCTP. The cDNA products were resolved on a 10% denaturing polyacrylamide gel. Reverse transcription is impeded by m^{6}A2058. cDNA synthesis terminates at G2056 and serves as a normalization reference.
