# Supplementary material for: Epitranscriptional m6A modification of rRNA negatively impacts translation and host colonization in Staphylococcus aureus
Source: PLoS Pathog. 2024 Jan 22;20(1):e1011968. doi: 10.1371/journal.ppat.1011968 (PMC10833563; doi:10.1371/journal.ppat.1011968)
Supplement: Attachment — Submitted filename: Point-by_Point_response.pdf [file ppat.1011968.s010.pdf]

We wish to thank the editors and 3 reviewers for their constructive comments and positive review. We have performed all the requested key experiments (Figure 1E, Figure S1D, Figure S2, Figure S4, and Table 1) and have fully addressed other minor concerns, including a lack of experimental details and presentation issues. Please refer to the marked-up document for all changes (highlighted in blue fonts)

Part I – Summary

Please use this section to discuss strengths/weaknesses of study, novelty/significance, general execution and scholarship.

Reviewer #1: The authors of the paper entitled "Epitranscriptional m^6A modification of rRNA negatively impacts translation and host colonization in Staphylococcus aureus" aimed to understand the bacterial physiological and gene expression effects of a rRNA modification that generates macrolide resistance in S. aureus. It is known from previous publications, including a couple from the senior corresponding author of this work, that changes in ribosome components, especially ribosomal proteins that generate antibiotic resistance can affect gene expression in bacteria as well as growth fitness. This work shows that methylation of nucleotides at rRNA that confer resistance to macrolides also affect gene expression and growth fitness. The authors used a series of ermBL and ermB mutant genes to enhance the presence of methylation at the 23S rRNA A2058 nucleotide of S. aureus. Bacteria carrying such methylation showed alteration in transcription and translation of several genes, corresponding as well with the incapacity of the cells to compete in vitro and in vivo with non modified cells. Despite that the main question of the work has been addressed before, the application of new methods (ribosome profiling) gives a new perspective to this important scientific area. The document is well written and the experimental designs are well constructed. The work opens avenues for further experimentation to understand the mechanism involved in these observations.
Re: We thank the reviewers for recognizing the significance of this study.

Reviewer #2: In their manuscript, Shields and coauthors studied the impact of 23S ribosomal methylation upon translation efficacy, colonization and infection. They focus on the methylation of A2058 on the 23S rRNA subunit which is responsible for antibiotic resistance to MLS. The authors show that the ermBL leader sequence is critical for the regulation of MLS resistance. Then, they did competition experiments using strains that have different markers (Tet or CcCl2) to show that hypermethylation of A2058 is detrimental for in vitro fitness or infection in a murine model in the absence of an antibiotic stress. They perform Ribo-seq and mRNA seq and described that many genes related to virulence, metabolism and information processing are downregulated in hypermethylated strain although a substantial number of genes is upregulated. At the end of their study, they used a nice dual-fluorescence reporter to determine translational output and accuracy in hypermethylated strain. The story is well-written, somehow short and provide additional information that goes beyond antibiotic, although the real impact of such study is not easy to appreciate. The story is well constructed but there are things that needs to be improved or better explained.
Re: We appreciate the comments. We have attempted to improve the clarity and presentation at our best. Additional new experiments were also included (see below).

Line 140: There is not many information regarding the $ermBL^{R7STOP}$-$ermB^{I75T/N100S}$ variant. In the experimental procedures, details regarding on how the authors obtained this variant is very limited. Then, I don't understand why the authors did not construct an $ErmBL^{WT}$-$ermB^{I75T/N100S}$ variant. In table 1, MIC are the same compared with $ermBL^{WT}$-$ermB^{WT}$ variant, except for SOL. Using the $ErmBL^{WT}$-$ermB^{I75T/N100S}$ variant would have clearly show that it can serve as a positive control for hypermethylation.
Re: We apologize for a lack information about the *$ermBL^{R7stop}$-$ermB^{I75T/N100S}$* variant. The *$ermB^{I75T/N100S}$* variant was obtained from a laboratory in vitro evolution experiment. We have included the experimental procedures under "Materials and Methods-strains" section. Indeed, the initial mutant allele that we had obtained from the in vitro evolution was the *$ermBL^{WT}$-$ermB^{I75T/N100S}$*. Due to its erythromycin-inducible *$ermBL^{WT}$* sequence, the *$ermBL^{WT}$-$ermB^{I75T/N100S}$* exhibits intermediate resistance to MLS antibiotics (between *$ermBL^{WT}$-$ermB^{WT}$* and *$ermBL^{R7stop}$-$ermB^{WT}$*) and is still sensitive to the newer generation

solithromycin (SOL). In contrast, *ermBL*$^{R7stop-}$*ermB*^{I75T}/^{N100S} is hyper-resistant to all tested MLS antibiotics. It appears that hyper-resistance against all MLS antibiotics requires a concerted action of (1) constitutive ErmB overexpression and (2) I75T/N100S mutations that either promotes methyltranferase activity or protein stability. Therefore, we consider *ermBL*$^{R7stop-}$*ermB*^{I75T}/^{N100S} a suitable hypermethylation control. This is also supported by our unpublished cryo-EM structure of ErmB-methylated ribosomes (in collaboration with Ada Yonath's lab), in which *ermBL*$^{R7stop-}$*ermB*^{I75T}/^{N100S} variant produces almost if not all dimethylated m$^6_2$A2058 ribosomes whereas other variants produce a mixture of mono-methylated m^{6}A2058 and dimethylated m$^6_2$A2058 ribosomes. We attempted to better characterize the methyltransferase activity of ErmB$^{I75T/N100S}$ protein but to this end we have not had a lot of success due to the recalcitrant insolubility of the recombinant protein when expressed in the *E. coli* host. We will be exploring an overexpression strategy in *S. aureus* and/or the closely related *B. subtilis* host.

Line 142: The authors mentioned an "extremely low basal level of methylation". It seems that there is actually nothing, or very similar that that of the negative control Y103A.
Re: We have corrected the sentence to read "has undetectable level of methylation".

The competitive fitness experiment is interesting, but why the authors did not try to test infection in murine model under erythromycin condition to verify if in vitro results are fully reproducible?
Re: Our focus was to examine a possible fitness loss of m^{6}A2058 strain WITHOUT any antibiotic selection. Since our in vitro competition experiments had already showed a clear growth advantage of m^{6}A2058 strain in the presence of low erythromycin (~1.5× MIC, Figure 2A), it would be unnecessary to sacrifice hundreds of mice (including optimizing dosage and duration for intraperitoneal injection) to prove something obvious. The cost associated with entire experiments is also very prohibitive for a small lab like ours.

Line 188: The authors used epifluorescence to study bacterial morphology. However, electron microscopy and measurement of cell wall thickness may have been more appropriate.
Re: We agree with the reviewer that epifluorescence microscopy does not have sufficient resolution. We performed transmission electron microscopy (TEM) and confirmed that cell wall thickness and ultrastructure are virtually unchanged in *S. aureus* strains carrying active and inactive ErmB. The new TEM results are now included in Figure S1, panel D.

The authors do not sufficiently describe the section related to RPF and mRNAseq. They do not provide any numbers regarding the actual number of genes that are downregulated and upregulated. It seems that there are more genes upregulated that downregulated. This information is really important. Although I can understand that they do not have an explanation regarding this increase, they cannot just state that many virulence genes are downregulated, which tend to send a message to there an overall decrease in gene expression. Additionally, why not doing a DEG analysis using DESeq2 for mRNAs?
Re: We apologize for the missing information. The numbers of downregulated (n=129) and upregulated (n=204) genes in translational efficiency (TE) are indicated in Figure 3C. We have now explicitly mentioned the numbers in the main text under "Results" section and the gene list is included in Supplementary Dataset 1. We also included a description and relevant citations of how RPKM was calculated using the featurecounts and edgeR under "Materials and Methods" section.

Figure 4: Why there is almost nothing on the lane ermBL$^-$-ermB$^-$ for Nuc2?
Re: Our original gel image might not have the appropriate contrast, leading to the notion that Nuc2 is not expressed in the *ermBL*$^-$-*ermB*$^-$. As shown in the revised Fig. 4 Nuc2 panel, the precursor and secreted form of Nuc2 are clearly visible in the *ermBL*$^-$-*ermB*$^-$ strain, albeit at a much lower level than other strains. Judging by a lower molecular weight Nuc2 signal (marked by #), we suspect that Nuc2 is more readily degraded in the *ermBL*$^-$-*ermB*$^-$ strain for a reason that we don't fully understand. We have included this speculation in the "Results" section.

Line 255: Based on results in WT, one could have imagined the level of Yfp should be lowered in all condition. Please comment.

Re: The levels of YFP are indeed slightly higher than we expected, but the degree of YFP/mcherry production in the mutant reporters (2- 8%) was comparable to the prototypical reporter constructs in *E. coli* cells (PMID: 28781237), suggesting that translational recoding and readthrough may be more pervasive than previously thought.

Reviewer #3 (received separately on 12-14-2023)
The manuscript is well written, the methods are sound, and the results are of obvious scientific merit. I fully support publishing this paper. There is only one typing error on page 10: dsyregulated - must be dysregulated.
Re: Thank you for the positive review.

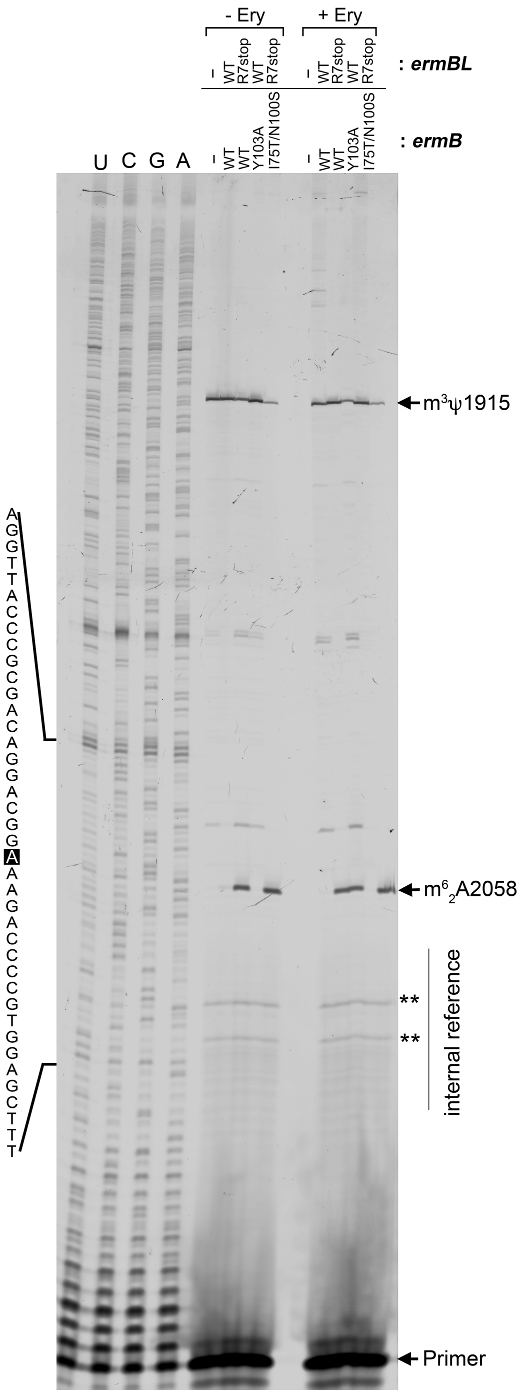

Part II – Major Issues: Key Experiments Required for Acceptance
Please use this section to detail the key new experiments or modifications of existing experiments that should be absolutely required to validate study conclusions.Generally, there should be no more than 3 such required experiments or major modifications for a "Major Revision" recommendation. If more than 3 experiments are necessary to validate the study conclusions, then you are encouraged to recommend "Reject".
Reviewer #1: To enhance the presentation of this work, this reviewer suggests the following:

Figure 1D. The primer extension signals above the A2028 methylation signal are not consistent with expectations. These weak signals are shown at ribosomes from cells without ermB cassettes; they are also shown in cells with the ermBLR7stop, but not with ermBL wild type under either + or - erythromycin. Are the amounts of rRNA similar per each sample? Would be great if the authors show somewhat that each lane reaction used the same amount of rRNA.
Re: The amount of rRNA in each sample is 250 ng, we have indicated the quantity in both the figure legend and the “Materials & Methods”. Weaker signal above the methylation site could sometimes (not always) be an indicator of halted reverse transcription. In the attached full gel image of Figure 1D, the m3-U1915 pseudouridylation signal clearly show that less extension occurs in the lanes containing m^{6}A2058. Other weaker signals above A2058 are reproducible throughout all of our primer extension experiments despite consistent use of equal rRNA template (250 ng). Varying readthrough of the reverse transcriptase could be the cause. Similar differential cDNA readthrough have been observed in other study (PMID: 33462493, Fig. 1e) and it was interpreted as possible nucleotide misincorporation or contamination. In our opinion, the signals (marked by **) below the methylation site are more faithfully reflecting the actual input. The label in Figure 1D has been revised accordingly.

Also, could the authors determine % of methylated rRNA by using their primer extension assays? Comparison between their data and previous observations (Gupta et al. Nat Commun. 2013;4:1984.) seems to be essential for their manuscript.
Re: We have followed the reviewer’s recommendation and repeated the primer extension with modified d(d)NTP concentrations. We show that the plasmid-encoded ErmC (Gupta et al. Nat Commun.

2013;4:1984.) and chromosomally located ErmB overexpression both achieve >50% methylation. The new data is included in Figure S2.

Regarding stability of ribosomes or ribosome synthesis: the ribosome sedimentation assays shown at Fig S2 could be used to calculate the amount of ribosomes per cell?
Re: We would like to clarify that the ribosomes in Fig S2 (now Figure S3) were harvested at the same cell density. Each density gradient also contains equal amount of input, which is 25 units Abs_{260nm} of RNA (equivalent of 1 mg RNA). Normalizing loading by RNA concentrations has been a gold standard in ribosome biology field. We are unsure how ribosome sedimentation profiles can be translated into cellular concentrations of ribosome without additional probing of ribosomal components with immunoblotting. We have indicated the amount of RNA input and cell density at the points of harvesting in the figure legend (see Figure S3).

Figure 1E. Is there any way that the authors could concentrate the protein sample to get a stronger signal?
Re: We have repeated the Western blot with higher protein load and have substituted the original panel with a new Figure 1E showing stronger ErmB signals.

Figure 2B. I suspect the authors might have done in vivo experiments with erythromycin as well. If such, would it be significant to show them as well? It might correlate with the in vitro data.
Re: We acknowledge that this is one of our experimental limitations. We have not done any animal work with the administration of erythromycin, which will involve tedious intraperitoneal injection that can cause significant distress and pain to the animals. Our original focus was to examine a possible fitness loss of m^6A2058 strain WITHOUT any antibiotic selection. Since our in vitro competition experiments had already showed a clear growth advantage of m^6A2058 strain in the presence of low erythromycin (~1.5× MIC, Figure 2A), it was unnecessary to sacrifice hundreds of mice to prove something obvious, aside from the cost associated with the entire experiments that is quite prohibitive for a small lab.

Lines 208-210: The authors wrote: “Local accumulation of ribosomes at specific codon positions of mRNA followed by sparse downstream ribosome density is indicative of ribosome stalling. Such a signature was not observed in either strain.” This reviewer is wondering if there are known gene sequences in S. aureus that produced such arrest signatures seen for example in other bacteria genes such as secM, tnaC, speFL, in E. coli, that could be used as positive controls of their method.
Re: Low GC gram-positive bacteria such as *S. aureus* do not carry known ribosome stalling peptides SecM, TnaC, SpeFL and VemP. Leader peptide-mediated translational or transcriptional attenuation is less well-studied in *S. aureus*. A homolog of *B. subtilis* ribosome arrest peptide MifM is also absent in *S. aureus*. However, we were able to show the known ribosome stalling event upstream of the *ilv-leu* operon (PMID: 29357354) and +1 frameshifting in the *prfB* gene, providing a proof-of-principle for our Ribo-seq. The ribosome density plots are presented in new Figure S4 (panels A-B).

Also, the authors might want to address the previous observations about the gene poxB (Gupta et al. Nat Commun. 2013;4:1984.)
Re: Unlike previous study (Gupta et al. Nat Commun. 2013;4:1984.), we did not observe ribosomal frameshift in *poxB* (a.k.a *cidC*) nor ribosome pausing in *tenA* (Figure S4, panels C-D). This inconsistency is largely due to the use of different *S. aureus* strains in both studies. We have included these comparisons in the “Discussion” section.

Figure 5B, could the authors suggest an explanation of why methylation of A2058 would affect the wild type YFP/mcherry ratio? Since it is a long peptide, it seems that the ribosome modification is either reducing YFP production or increasing mCherry production. This reviewer was expecting to see the same ratio if both ORFs were affected equally. Could it be a ribosome arrest prior to reaching the YFP ORF?
Re: The reviewer is likely correct that m^6A2058 ribosomes may be less efficient in both translational initiation and elongation. Our reporter assays unfortunately could not distinguish the two scenarios. We

do not know if ribosome stalling could occur preceding the *yfp* coding region since no known ribosome arrest sequence, e.g., tandem positively charged residues and polyprolines etc. are apparent in the upstream *mcherry.* We had attempted to purify m^6A2058 ribosome and test these possibilities in a *S. aureus* in vitro translation system coupled with primer extension. The results were inconclusive as we were not able to obtain homogenous population of m^6A2058 ribosomes (i.e., only 50-60% of ribosomes are dimethylated, Figure S2). Enrichment-purification of the m^6A2058 ribosomes is an ongoing effort.

Also, for the case of WT and TAG: could the authors suggest that reduction seen in this figure could be instead of “slight”, 50 and 25% reduction respectively?
Re: We apologize for the confusion. The y-axis represents the Yfp/mCherry ratio with WT reporter reference set as 1 (100%), thus the stop codon reporters contain only 5%-2.5% of fluorescence ratio relative to the WT reporter. The “slight” reduction was meant to describe the comparison between the inactive and active ErmB strains.

Reviewer #2:
Line 140: There is not many information regarding the ermBLR7STOP-ermBI75T/N100S variant. In the experimental procedures, details regarding on how the authors obtained this variant is very limited. Then, I don’t understand why the authors did not construct an ErmBLWT-ermBI75T/N100S variant. In table 1, MIC are the same compared with ErmBLWT-ermBWT variant, except for SOL. Using the ErmBLWT-ermBI75T/N100S variant would have clearly show that it can serve as a positive control for hypermethylation.
Re: This is a duplicated question. See response on Page 1.

Line 188: The authors used epifluorescence to study bacterial morphology. However, electron microscopy and measurement of cell wall thickness may have been more appropriate.
Re: This is a duplicated question. See response on Page 2.

The authors do not sufficiently describe the section related to RPF and mRNAseq. They do not provide any numbers regarding the actual number of genes that are downregulated and upregulated. It seems that there are more genes upregulated that downregulated. This information is really important. Although I can understand that they do not have an explanation regarding this increase, they cannot just state that many virulence genes are downregulated, which tend to send a message to there an overall decrease in gene expression. Additionally, why not doing a DEG analysis using DESeq2 for mRNAs?
Re: This is a duplicated question. See response on Page 2.

Part III – Minor Issues: Editorial and Data Presentation Modifications
Please use this section for editorial suggestions as well as relatively minor modifications of existing data that would enhance clarity.

Reviewer #1: Line 271, consider changing the following: “changes are due to an indirect” to “changes could be due to an indirect”
Re: We have rephrased as recommended.

Line 287, change “dsyregulated” to “dysregulated”
Re: corrected.

Table 1, could be better for the viewer to change “nd” to “-”. The table seems busy.
Re: We have now included the full set of MIC data for the *ermB*$^{I75T/N100S}$ variant, the table is no longer as busier as the original version, thus changing “nd” to”-“ is unnecessary.

Reviewer #2: Line 142: The authors mentioned an “extremely low basal level of methylation”. It seems that there is actually nothing, or very similar that that of the negative control Y103A.
Re: This is a duplicated question as on Page 2. We have reworded to read “undetectable level of methylation”.

The competitive fitness experiment is interesting, but why the authors did not try to test infection in murine model under erythromycin condition to verify if in vitro results are fully reproducible?
Re: This is a duplicated question. See response on Page 4.

Figure 4: Why there is almost nothing on the lane ermBL^{-}-ermB^{-} for Nuc2?
Re: This is a duplicated question. See response on Page 2.

Line 255: Based on results in WT, one could have imagined the level of Yfp should be lowered in all condition. Please comment.
Re: This is a duplicated question. See response on Page 2-3.

Reviewer #3 (received separately on 12-14-2023):
Page 10: dsyregulated - must be dysregulated.
Re: corrected.
